# Macromolecular and elemental composition analysis and extracellular metabolite balances of *Pichia pastoris *growing at different oxygen levels

**DOI:** 10.1186/1475-2859-8-65

**Published:** 2009-12-09

**Authors:** Marc Carnicer, Kristin Baumann, Isabelle Töplitz, Francesc Sánchez-Ferrando, Diethard Mattanovich, Pau Ferrer, Joan Albiol

**Affiliations:** 1 Departament d'Enginyeria Química, Escola d'Enginyeria, Universitat Autònoma de Barcelona, 08193-Bellaterra (Cerdanyola del Vallès), Spain; 2 Departament de Química, Universitat Autònoma de Barcelona, 08193-Bellaterra (Cerdanyola del Vallès), Spain; 3 Department of Biotechnology, BOKU-University of Natural Resources and Applied Life Sciences, Vienna, Austria; 4 School of Bioengineering, University of Applied Sciences FH-Campus Wien, Vienna, Austria; 5 Current address: Ludwig Boltzmann Institute for Cancer Research (LBI-CR), Vienna, Austria

## Abstract

**Background:**

Analysis of the cell operation at the metabolic level requires collecting data of different types and to determine their confidence level. In addition, the acquired information has to be combined in order to obtain a consistent operational view. In the case of *Pichia pastoris*, information of its biomass composition at macromolecular and elemental level is scarce particularly when different environmental conditions, such as oxygen availability or, genetic backgrounds (*e.g*. recombinant protein production *vs*. non production conditions) are compared.

**Results:**

*P. pastoris *cells growing in carbon-limited chemostat cultures under different oxygenation conditions (% O_2 _in the bioreactor inlet gas: 21%, 11% and 8%, corresponding to normoxic, oxygen-limiting and hypoxic conditions, respectively), as well as under recombinant protein (antibody fragment, Fab) producing and non-producing conditions, were analyzed from different points of view. On the one hand, the macromolecular and elemental composition of the biomass was measured using different techniques at the different experimental conditions and proper reconciliation techniques were applied for gross error detection of the measured substrates and products conversion rates. On the other hand, fermentation data was analyzed applying elemental mass balances. This allowed detecting a previously missed by-product secreted under hypoxic conditions, identified as arabinitol (*aka*. arabitol). After identification of this C5 sugar alcohol as a fermentation by-product, the mass balances of the fermentation experiments were validated.

**Conclusions:**

After application of a range of analytical and statistical techniques, a consistent view of growth parameters and compositional data of *P. pastoris *cells growing under different oxygenation conditions was obtained. The obtained data provides a first view of the effects of oxygen limitation on the physiology of this microorganism, while recombinant Fab production seems to have little or no impact at this level of analysis. Furthermore, the results will be highly useful in other complementary quantitative studies of *P. pastoris *physiology, such as metabolic flux analysis.

## Background

The operation of living cells can be viewed as a complex network of interacting biomolecules. In order to gain a deeper understanding of the cell's response to different environmental conditions, a number of high throughput analyses are nowadays performed, covering different cellular levels such as the genome, transcriptome, proteome, metabolome and fluxome [1-3]. Metabolic flux analysis (MFA), provides a highly informative view of the physiological cell status under a given environmental condition or genetic background. Genome-scale *in silico *metabolic models are nowadays being built for a number of microorganisms to this purpose [4,5]. Validation and practical application of such complex models require obtaining reliable experimental data on a number of metabolic fluxes, among which those related to biosynthesis precursors for cell constituents play a key role [6,7]. Considering that cells are made up of different biopolymers and macromolecules, knowledge of their composition and quantity becomes essential for determination of the metabolic fluxes of biosynthetic precursors, as well as for any other metabolic or energetic analysis. For instance, determination of metabolic fluxes from the measured ^13^C isotopomer distribution of proteinogenic amino acids [8] requires to measure not only the external metabolic fluxes of the cell, but also the amino acid composition of the proteins being produced so that drain of biosynthetic precursors towards biomass synthesis can be properly taken into account. Availability of such molecular compositional data is scarce or inexistent for a specific strain or species and growth condition, particularly in non-model organisms as *P. pastoris*. Previous application of the above mentioned methodologies on *Pichia pastoris *[9,10] has mainly relied on available compositional data from a related species such as *S. cerevisiae *[3].

Determination of biomass molecular composition usually relies on application of different analytical techniques for the range of biochemical compounds considered, each one with its own sensitivity, interferences and confidence level; moreover, some of them providing redundant information. Application of the numerical techniques for metabolic flux analysis requires the quantitative determination of a consistent biomass composition. For example, percentages of macromolecular components analysed by different methods must ideally combine to 100% and the addition of their elemental compositions must combine to give the measured elemental composition. Also, confidence intervals for all the elements and components have to be calculated. To such purpose, statistical techniques have been developed [11] allowing to obtain the best estimation of such consistent biomass composition and the corresponding confidence intervals. Furthermore, such consistency should be extended to the measured input/output fluxes of the cells in order to verify that the system operates as expected [12,13].

In this study, a consistent description of the biomass composition of *P. pastoris *has been obtained for different oxygenation conditions, as well as under recombinant protein producing and non-producing backgrounds. In addition, a consistency analysis of the input and output extracellular metabolic fluxes has been used to identify the production of an unexpected by-product, which has been subsequently identified as arabinitol. Besides, multilevel analyses using *P. pastoris*-specific DNA arrays and proteomic techniques have been performed in parallel to this baseline study [14].

## Results and discussion

In this study, the differences in biomass composition are evaluated for two different *P. pastoris *strains, namely the X-33-derived strain expressing the human antibody fragment Fab 3H6 [15] and a control X-33 strain transformed with the corresponding mock expression vector. For these two strains, three different oxygenation conditions (normoxic, oxygen-limited and hypoxic, corresponding to O_2_, concentrations in the bioreactor inlet gas of about 21%, 11% and 8% of O_2_, respectively) were assayed in carbon-limited continuous cultures using glucose as carbon source. As previsouly described [16], *P. pastoris *shows a fully respirative metabolism under normoxic conditions, a shift towards respirofermentative metabolism can be observed under oxygen-limiting conditions, and a clearly respirofermentative metabolism is shown under hypoxic conditions. For each case, the different biomass composition analyses described in the materials and methods section were performed. At this point, the different biomass chemical analyses can be considered at two levels, resulting either in an elemental or a molecular description of the biomass constituents. The variations and similarities observed at these levels were further used to propose a consensus composition for each case.

### Molecular biomass composition

At the molecular level, and for simplicity, the biomass composition is usually described in terms of major groups of biomass constituents, namely proteins, carbohydrates, lipids, DNA and RNA [13]. In some cases, each of these groups is further analyzed for their major constituents. This procedure allows determining, not only their mass fractions and consensus global carbon molecular formula, but also which particular precursors and corresponding amounts are required for their biosynthesis.

### Proteins and amino acid content

**Figure 1:**
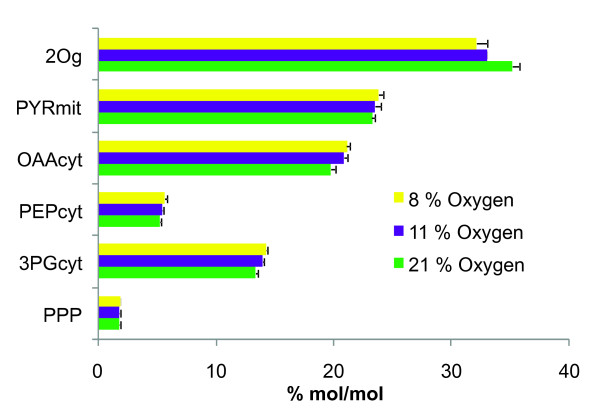
**Amount of key precursors required for protein generation at each experimental condition**. Using the average amino acid compositional data provided in Table 2, a calculation of the amounts of precursors consumed for protein generation was done. Nomenclature: α-keto-glutarate: 2Og, mitochondrial pyruvate: PYRmit, cytosolic oxalacetate: OAAcyt, cytosolic phosphoenolpyruvate: PEPcyt, cytosolic 3-phophoglycerate: 3PGcyt, pentose phosphate pathway: PPP.

Proteins are one of the major constituents of the biomass, usually representing around 50% of the dry weight of a microorganism [13]. Consequently, detailed knowledge of their composition is important for any metabolic and energetic calculations. As there are virtually no data on the amino acid composition of *P. pastoris *available, determination of this composition was performed for both strains at each of the tested oxygenation condition, as shown in Table 1. These analyses revealed that, for a given oxygenation condition, differences between the control and the Fab-expressing strain were, for most of the amino acids, below 9%. Some exceptions were found for cysteine (for which, one of the analysis resulted in amounts below detection limits), ornithine, methionine and valine. Nevertheless, these differences were apparently not correlated to changes in experimental conditions (oxygenation level or Fab production). Therefore, it was considered that the control and Fab-expressing strains did not show significant differences in their amino acid composition for any of the oxygenation conditions. Consequently, average amino acid composition values of *P. pastoris *(shown in Table 2) for each oxygenation condition were considered for further compositional data calculations. Notably, values given in Table 2, suggest that some amino acids (e.g. Glx, Orn, Arg) appeared to follow a consistent tendency to decrease as oxygen availability was reduced, while others seemed to remain at the same level (e.g. Thr, Trp, His, Met). Nevertheless, considering the standard deviations of the analysis (see Materials and Methods section), further experimental data would be required to confirm these tendencies. The amount of precursors consumed to produce cell proteins with the measured composition (mols of each amino acid *per *100 mols of amino acids), shown in Figure 1, was calculated taking into account the main *P. pastoris *biosynthetic routes for the amino acids synthesis; these were revised on the basis of its recently published genome sequence [17,18]). Grouping of amino acids, according to their common precursor in the central carbon metabolism (Figure 1), revealed that the amino acids precursor requirements showed a correlation with the variation of oxygen availability, with the exception of the precursors consumed from the pentose phosphate pathway. Following this observation, it was considered that those variations would have an impact on the calculated metabolic fluxes and, therefore, it was decided to adopt a different biomass composition for each of the three oxygenation conditions. In addition, data shown in Tables 1 and 2 reveal that the measured amino acid composition for *P. pastoris *was significantly different to the previously published amino acid composition of *S. cerevisiae *[11]. Interestingly, Orn presents several fold changes in terms of relative amounts between the two yeasts, while Arg, Ile, Leu, Val, Gly, Met and Ser show relative abundance values with a more than 10% variation from the *S. cerevisiae *data. These results justify the adoption of a specific amino acid composition for *P. pastoris *instead of the previously available compositional data from *S. cerevisiae*.

**Figure 2:**
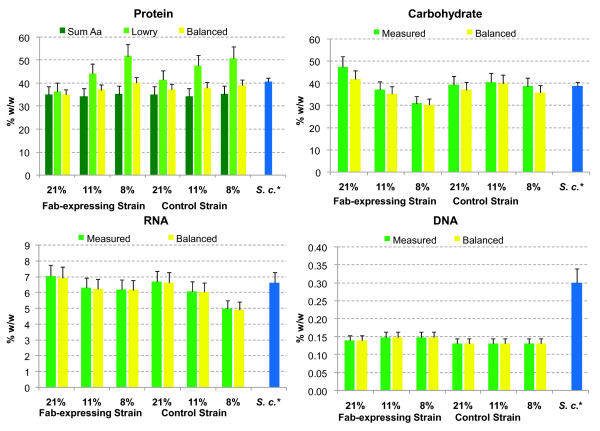
**Summary of the macromolecular biomass composition results obtained for the different experimental conditions and the final reconciled value, Top left: **Protein content, given as percentage of dry weight, measured using the Lowry method and calculated as the sum of extracted amino acids (A.a.), together with its reconciled value. **Top right:** Carbohydrate content % dry weight together with its reconciled value. **Bottom left:** RNA content given as percentage of dry weight together with its reconciled value. **Bottom right:** DNA content given as percentage of dry weight together with its reconciled value. S.c.: *S. cerevisiae*. * Data from Lange and Heijnen 2001 [11] for *S. cerevisiae *in blue bars.

**Figure 3:**
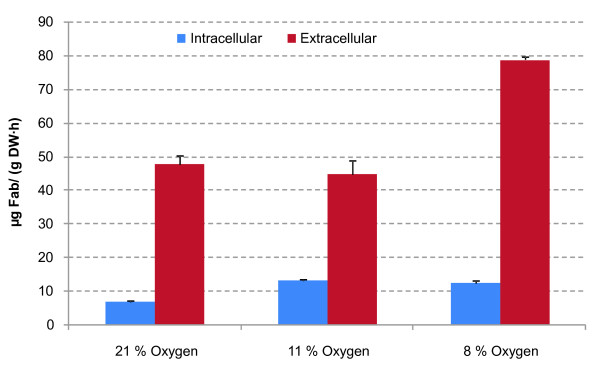
**Effect of decreasing oxygen supply on Fab heterologous protein production**. Measured Fab content in soluble cell extracts and in the extracellular medium of chemostat cultures of the Fab-producing strain growing under the different oxygenation conditions.

Total cell protein content (Figure 2 (top left)) was measured using two different methods. A first approximation was based on the sum of amounts of each amino acid obtained in the amino acid compositional analysis. Since this analytical technique involves an acid hydrolysis step of cell protein (see Materials and Methods section), it was expected that the value obtained by adding all the amino acids amounts should be on the lower margin. In the second approximation, the protein content of the biomass was measured using the widely used Lowry method, using bovine serum albumin as standard. The different values obtained and the standard deviation of the different analytical approaches made it difficult to calculate a proper value for the protein content for each case. Therefore, total protein content values given by the two methods, together with their confidence intervals were used in the data reconciliation step. This procedure allows calculating the total protein content that best agrees with all the information available. The reconciled values indicated an increase in the global protein content correlated with the oxygen limitation (see below: Reconciled biomass composition). In addition, the amount of intracellular and extracellular heterologous protein was measured in the Fab-expressing strain. Figure 3 shows that the amount of extracellular Fab increases when the oxygen concentration in the inlet gas decreases down to about 8%, in accordance with previous results [14]. At the same time, intracellular Fab content (which can also be considered as part of the total protein content) also increased significantly when reducing the oxygen availability. Nevertheless, further detailed studies are required in order to assess the potential impact of Fab production on total protein content and amino acid composition.

**Table 1:** Amino acid composition of the whole protein extract for all the conditions and strains tested

	***Pichia pastoris***						
		
**% mol/mol**	**Fab-expressing Strain**			**Control strain**			Saccharomyces cerevisiae *
			
	**21%**	**11%**	**8%**	**21%**	**11%**	**8%**	
		
Asx	8.82	9.78	9.59	8.78	9.51	9.78	9.28
Thr	5.88	5.98	5.78	5.77	5.90	6.16	5.57
Ser	6.26	6.65	6.73	6.43	6.76	6.90	5.33
Glx	17.81	16.24	15.64	18.55	16.19	14.33	15.48
Pro	3.83	4.08	4.47	3.67	4.00	4.76	4.22
Gly	6.86	7.34	7.42	7.12	7.28	7.53	8.89
Ala	10.40	9.92	10.13	10.68	9.74	9.88	9.77
Val	5.88	6.05	6.09	5.58	6.78	6.16	7.33
Cys	0.15	0.15	0.14	b.d.	0.22	0.13	0.14
Met	0.79	0.67	0.78	0.77	0.80	0.70	1.14
Ile	4.64	4.68	4.76	4.12	4.45	4.70	5.89
Leu	6.96	7.26	7.46	6.99	7.23	7.94	8.01
Tyr	2.16	2.22	2.27	2.13	2.21	2.47	1.96
Phe	3.20	3.30	3.33	3.03	3.21	3.37	3.76
Orn	1.04	1.02	0.86	1.53	1.21	0.93	0.24
Lys	6.41	7.07	6.93	6.33	7.03	6.93	6.57
His	1.89	1.87	1.87	1.79	1.78	1.89	1.93
Trp	1.40	1.40	1.40	1.40	1.40	1.40	1.96
Arg	7.04	5.72	5.76	6.74	5.68	5.45	3.86

Amino acid composition measured for the different experimental conditions (% O_2 _in the inlet gas: 21, 11, 8) and for the different strains. Data given as percentage of each amino acid in the total pool of amino acids. * Data from Lange and Heijnen 2001 [11] for *S. cerevisiae*. Glx represents the pair Glu/Gln. Asx represents the pair Asp/Asn. b.d: below detection limit.

**Table 2:** Average amino acid composition of the whole protein extract between the two strains for the different experimental conditions tested

	***Pichia pastoris***			Saccharomyces cerevisiae *
**%** **mol/mol**	**21%**	**11%**	**8%**	
		
Asx	8.67	9.50	9.55	9.28
Thr	5.74	5.85	5.88	5.57
Ser	6.25	6.61	6.72	5.33
Glx	17.91	15.98	14.76	15.48
Pro	3.69	3.98	4.55	4.22
Gly	6.89	7.21	7.36	8.89
Ala	10.39	9.69	9.86	9.77
Val	5.65	6.32	6.03	7.33
Cys	0.15	0.18	0.13	0.14
Met	0.76	0.73	0.73	1.14
Ile	4.32	4.50	4.66	5.89
Leu	6.87	7.14	7.59	8.01
Tyr	2.11	2.18	2.33	1.96
Phe	3.07	3.21	3.30	3.76
Orn	1.27	1.10	0.88	0.24
Lys	6.28	6.94	6.83	6.57
His	1.81	1.80	1.86	1.93
Trp	1.38	1.38	1.38	1.96
Arg	6.79	5.62	5.52	3.86

Amino acid composition measured for the different experimental conditions (%O_2 _in the inlet gas: 21, 11, 8). Data given as percentage of each amino acid in the total pool of amino acids. * Data from Lange and Heijnen 2001 [11] for *S. cerevisiae*. Glx represents the pair Glu/Gln. Asx represents the pair Asp/Asn.

### RNA content

The results obtained for RNA content are shown in Figure 2 (bottom left). In this case, the control strain seemed to show a decrease in the RNA content as the oxygen availability was reduced, while in the Fab-expressing strain this effect was only apparent for the change from normoxic (21% O_2_) to the lower oxygenation conditions whereas the RNA content in cells grown under oxygen limiting and hypoxic conditions remained similar.

Nevertheless, considering that RNA levels are expressed in relative terms (% weight RNA/% dry cell weight), these variations do not appear to be relevant when compared to the corresponding variations observed for the major cell components (e.g. cell protein). On the other hand, when comparing the control strain with the Fab-expressing strain at the same growth conditions, the RNA levels appeared to be generally slightly higher in the Fab-expressing strain. Nevertheless, such differences were not statistically significant for all oxygenation conditions. Notably, the relative decrease in RNA content under reduced oxygen availability conditions seemed to be less pronounced for the Fab-expressing strain. One might speculate that the increase in heterologous protein production under hypoxic conditions could result in higher RNA levels than in the control strain. Overall, the average value of all RNA levels measured for *P. pastoris*, appeared to be similar to those available from *S. cerevisiae *[11].

### Carbohydrate content

Figure 2 (top right), shows the cell's total carbohydrate content measured for both strains grown under the three tested oxygenation conditions. The control strain seemed to present fairly constant carbohydrate levels under all oxygenation conditions, which as an average, were comparable to the *S. cerevisiae *levels [11]. However, the Fab-expressing strain showed a significant decrease in its relative carbohydrate content as the oxygen availability decreased. Notably, this decrease was coherent with the higher increase in the relative total protein content and the lower decrease in RNA content in the Fab-expressing strain than in the control strain as the oxygen availability was reduced.

**Figure 4:**
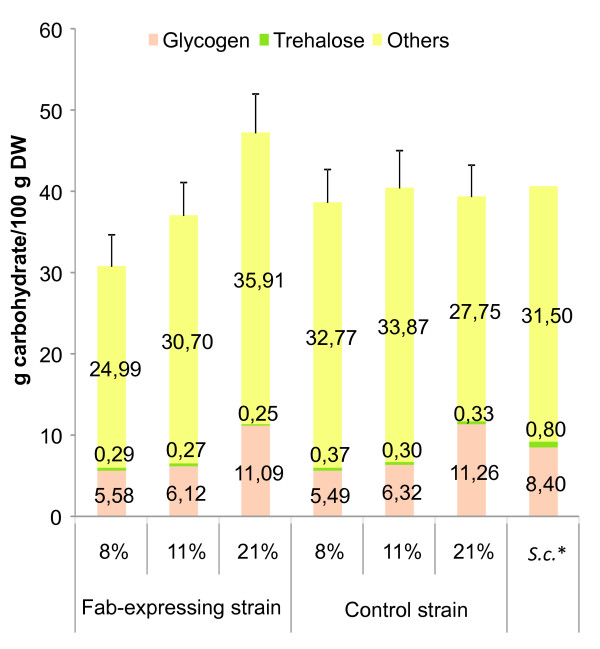
**Principal carbohydrates**. Total carbohydrate, glycogen and trehalose amounts measured for each strain and oxygenation condition. * Data from Forster et al. 2003 [3] for *S. cerevisiae*.

Moreover, although the total carbohydrates content changed slightly, glycogen relative amounts were different depending on the oxygenation conditions, showing a significantly higher value in cells grown under normoxic conditions than in the oxygen-limited and hypoxic ones (Figure 4). These results could be explained by the need to secrete differently reduced carbon compounds to the media in limited-oxygen and hypoxic conditions to maintain the redox balance inside the cell (see Metabolite mass balance section) Interestingly, one of the glycogen synthases (Gsy1) from *S. cerevisiae *appears to be regulated by Rox1, a transcriptional factor that regulates responses to oxygen [19], so one could speculate that glycogen metabolism in *P. pastoris *is also regulated at the level of gene expression by an environmental stressor such as hypoxia. On the other hand, the trehalose values did not show any clear tendency when varying the oxygenation degree or the strain genetic background.

### DNA content

As shown in Figure 2 (bottom right), the measured DNA content of *P. pastoris *was lower than the amounts previously reported for *S. cerevisiae *[11]. This might partially reflect the smaller size of the *P. pastoris *genome (haploid set of 4 chromosomes resulting in 9.43 Mbp [17]) compared to the one of *S. cerevisiae *(haploid set of 16 chromosomes resulting in 12 Mbp [20]). On the other hand, industrial strains of *S. cerevisiae *are usually found under diploid (or even polyploid) state [21], while *P. pastoris *is in haploid state [22]. Combination of both factors could explain the differences among the measured DNA content of the two species.

### Lipid content

With respect to the lipid fraction content, an effort was done to quatify the total lipid content of the biomass as well as determining semi-quantatively the major components of this fraction. However, the methodology employed showed low levels of total lipid recovery. Therefore, the data obtained was only used to determine the major components of the lipid family, whereas the total amount was instead estimated during the reconciliation procedure (see below). The total lipid content of the biomass calculated by the reconciliation procedure appeared to be close to the data available for *S. cerevisiae *[11].

On the other hand, the major lipid components identified in the thin layer chromatography were triacylglycerides (TAG), ergosterol (ERG), phosphatidylcholine (PC), and phosphatidylethanolamine (PE) in order of abundance. The triacylglycerides relative amounts seemed to increase when the oxygen availability decreased while ergosterol seemed to decrease. Nevertheless, the technique used for this analysis did not allow for a very precise quantification of each lipid component. Therefore, the average percentage of abundance, for each of the major lipid components, was taken to calculate the lipid C-mol formula: triacylglycerides (57.2% w/w), ergosterol and ergosterol esters (26.8% w/w), phosphatidylcholine (10.0% w/w), phosphatidylethanolamine (5.2% w/w), phosphatidylinositol + phosphatidylserine (0.4% w/w), cardiolipin (0.3% w/w) and phosphatidic acid (0.1% w/w).

### Elemental biomass composition

As explained above, besides the macromolecular composition of the biomass, its content of major chemical elements can be also considered. Usually, the elements that are taken into account are those that add up to nearly 90% of the dry weight, namely C, H, O, N, P, and S. In this study, only C, H, N, S and ash content were measured (see Additional file 1). For the reconciliation procedure, P was included into a composite compound which also included ashes. Therefore, the reconciliation procedure calculated the oxygen content that fulfilled the global balance taking into account all the available information (see below: Reconciled biomass composition).

### Consistency check and data reconciliation

As mentioned in the Introduction section, in order to perform detailed metabolic flux analyses calculations, it is necessary to verify the consistency of the data obtained and, when different kinds of complementary or redundant data is available, to obtain the best estimation of the studied system using common data reconciliation techniques [23]. This procedure was performed in two steps. In the first one, the different biomass compositional data available was used to obtain a reconciled biomass composition. In the second step, the consistency check of the measured substrate consumption together with the biomass and products generated was performed.

### Reconciled biomass composition

Calculation of a reconciled biomass composition from the biomass compositional data described above was performed using the maximum likelihood method as described by Lange and Heijnen [11]. In short, the method allows the calculation of the best estimation of the biomass composition by solving a linear system which takes conservation laws of elemental and mass balances into account. The obtained experimental data is considered using a weight proportional to the confidence of the analysis.

For the reconciliation procedure, the elemental composition of the macromolecular compounds, in terms of a C-mol formula, of each biomass component was calculated as follows: Protein elemental composition was calculated from the measured relative abundance of each amino acid in *P. pastoris *samples. The elemental composition of the carbohydrate component was considered to be a glucose polymer. The elemental composition of lipids was calculated as an average of the major compounds identified. DNA elemental composition was calculated from the percentage of its nucleotide content in *P. pastoris *[17]. RNA elemental composition was calculated as proposed by Stephanopoulos [13]. Other components, such as metals, H_2_O and sulphate were included in an identical way as described for *S. cerevisiae *in Lange and Heijnen [11] (See Additional file 2 for the C-mol formulas).

Table 3 shows the values for the biomass composition obtained after the reconciliation procedure was applied. The reconciliation procedure provides the best estimation of the biomass composition, including the lipid content or the oxygen values that were not measured. The biomass composition obtained this way is also consistent from the viewpoint that all the macromolecular elements considered add to 100% and the chemical elements composing them also add up to 100%. Moreover, the standard deviation of the estimated values is given, as is of utmost importance for any further numerical treatment. Overall, the calculated biomass compositional values show that, as the oxygen availability is decreased, there is a slight increase in the protein content of the biomass. Also, the calculated data indicates an increase in the total lipid content, which has a higher degree of reduction than the proteins or the carbohydrates. Nevertheless, taking into consideration the standard deviation calculated for the lipids, the confidence for the calculated values is low and, therefore, more data will have to be obtained in the future to confirm the indicated tendency.

**Table 3:** Biomass composition calculated after the reconciliation procedure

	**Fabb-expressing strain**						**Control strain**					
		
	**21%**		**11%**		**8%**		**21%**		**11%**		**8%**	
		
	**% w/w**	**sd**	**% w/w**	**sd**	**% w/w**	**sd**	**% w/w**	**sd**	**% w/w**	**sd**	**% w/w**	**sd**
	
Protein	34.9	2.3	37.0	2.4	40.0	2.6	37.0	2.4	37.8	2.5	38.9	2.6
Carbohydrate	41.7	4.0	35.0	3.3	30.1	2.9	36.9	3.5	39.7	4.0	35.6	3.5
Lipid	6.6	3.4	9.8	3.2	10.3	3.2	6.2	3.3	7.8	3.5	8.7	3.3
RNA	6.9	0.7	6.2	0.6	6.1	0.6	6.6	0.7	6.0	0.6	4.9	0.5
DNA	0.14	0.01	0.15	0.01	0.15	0.01	0.13	0.01	0.13	0.01	0.13	0.01
	
SO_4_	0.2	0.2	0.2	0.2	0.1	0.2	0.3	0.3	2.7	3.5	0.2	0.2
H_2_O	3.2	2.5	5.2	2.4	6.8	2.2	6.3	2.4	0.2	0.2	5.0	2.4
Metals	6.4	0.4	6.4	0.4	6.4	0.4	6.4	0.4	5.6	1.2	6.5	0.4
	
C	44.3	1.4	44.6	1.4	44.4	1.4	43.0	1.4	45.6	1.2	44.7	1.4
H	6.2	0.2	6.5	0.2	6.6	0.2	6.3	0.2	6.0	0.2	6.4	0.2
N	6.7	0.4	6.9	0.4	7.4	0.4	6.9	0.4	7.0	0.4	7.0	0.4
O	35.6	1.4	34.8	1.4	34.3	1.4	36.4	1.4	34.1	1.3	34.6	1.4
S	0.2	0.1	0.2	0.1	0.1	0.1	0.2	0.1	0.2	0.1	0.2	0.1
	
Ashes	7.1	0.4	7.1	0.4	7.2	0.4	7.1	0.4	7.1	0.4	7.1	0.4

Biomass composition in terms of macromolecular and elemental compounds for the different experimental conditions (% O_2 _in the inlet air 21, 11, 8).

The reconciliation procedure also calculates the major chemical elements contained in the biomass, including the non measured oxygen. This allows calculating a biomass carbon elemental formula, shown in Table 4. It can be observed that the ratio H:O and the biomass degree of reduction increase as the oxygen availability is decreased. This is in agreement with the accumulation of more reduced biomass components, for instance, an increase in lipid content. This effect is apparently more pronounced in the Fab-producing strain. The resulting reconciled biomass composition was used in the rest of the calculations.

**Table 4:** Biomass C-molecular formula calculated after the reconciliation procedure

	*C-mol Biomass formula*		C:N Ratio	H:O Ratio	**(**γ**)**
Fab Expressing Strain	21% oxygen	C H_1.665 _N_0.134 _O_0.602 _S_0.0039_	7.45	2.77	4.11
	11% oxygen	C H_1.731 _N_0.137 _O_0.585 _S_0.0015_	7.30	2.96	4.18
	8% oxygen	C H_1.775 _N_0.144 _O_0.579 _S_0.0012_	6.94	3.06	4.20

Control Strain	21% oxygen	C H_1.761 _N_0.143 _O_0.636 _S_0.0018_	7.02	2.77	4.09
	11% oxygen	C H_1.585 _N_0.135 _O_0.560 _S_0.0016_	7.39	2.83	4.08
	8% oxygen	C H_1.726 _N_0.138 _O_0.581 _S_0.0014_	7.26	2.97	4.17

S. cerevisiae *		C H_1.748 _N_0.148 _O_0.596 _S_0.0018_	6.76	2.93	4.22

Elementary composition of biomas, expressed as C-molecular formula, for the different strains and experimental conditions (% O_2 _in the inlet air: 21, 11, 8). C:N carbon:nitrogen ratio; H:O hydrogen:oxygen ratio. γ: reduction degree of the biomass. * Data from Lange and Heijnen 2001 [11] for *S. cerevisiae*.

### Metabolite mass balances

One of the key points in any fermentation study is to validate the measured consumption of substrates and generation of products. This step has been performed following the well established statistical procedures for the purpose [12,13,24-27], which are similar to the one already followed for the reconciliation of the biomass composition data. The biomass C-molecular formula calculated in the previous step is used in this procedure. Then, the χ^2^-distributed consistency index h has been calculated for the reconciled data to test for the presence of gross errors according to a well established procedure (Wang [23], van der Heijden [25-27] and Stephanopoulos [13]).

In a first approximation, it was considered that biomass and CO_2 _generation under normoxic conditions resulted from the glucose and oxygen consumption, while reduction in the oxygen availability resulted in the generation some by-products such as pyruvate, glycerol, ethanol and citrate. As can be observed in Table 5, a carbon balance performed in both strains for the normoxic conditions (21% of oxygen in the inlet air), indicated that most of the carbon was taken into account, as there was only a mismatch of 6% and 1% respectively, well in agreement with the precision of the analyses. However, in the case of cultivations under oxygen-limiting and hypoxic conditions, the carbon balance showed a significant mismatch, suggesting that a compound was most probably missing. In addition, observation of the HPLC chromatograms of cleared supernatants from these cultivations (Additional file 3) showed an unidentified peak which increased as oxygen limitation was more severe. The compound corresponding to this peak was isolated and further analysed by LC-MS, resulting in the identification of a compound of a molecular mass of 197 which taking into account the effect of the reacting eluent (formic acid, 46 m/z) results in a metabolite of 151 g/mol.

**Table 5:** Measured substrates and products consumed/produced at steady state in each experimental condition

	Fab-expressing strain						Control strain					
		
mmol/gDW·h	21%		11%		8%		21%		11%		8%	
		
		sd		sd		sd		sd		sd		sd
Glucose	-0.99	0.05	-1.33	0.07	-1.74	0.09	-0.99	0.05	-1.32	0.07	-1.92	0.10
OUR	-2.12	0.17	-1.57	0.08	-0.54	0.10	-2.12	0.17	-1.66	0.33	-0.28	0.01
CER	2.37	0.19	2.03	0.10	1.65	0.08	2.37	0.19	2.09	0.42	1.35	0.27
Biomass	3.01	0.30	3.71	0.37	3.73	0.19	3.59	0.36	3.70	0.37	3.76	0.38
Ethanol	-	-	0.33	0.02	1.00	0.05	-	-	0.31	0.02	1.16	0.06
Glycerol	-	-	0.01	0.001	0.02	0.001	-	-	0.02	0.001	0.02	0.001
Citrate	0.03	0.002	0.02	0.001	0.04	0.002	0.01	0.001	0.02	0.001	0.05	0.02
Arabinitol	-	-	0.21	0.01	0.42	0.02	-	-	0.10	0.01	0.40	0.13
Pyruvate	-	-	0.06	0.003	0.09	0.005	-	-	0.06	0.002	0.12	0.01

% C Balance Error excluding arabinitol	6.4%		15.7%		23.9%		1.3%		14.5%		29.3%	

% C Balance Error including arabinitol	6.4%		2.5%		3.7%		1.3%		8.2%		11.9%	

Substrate consumption, biomass and metabolites production rates, for each strain and the different experimental conditions (% O_2 _in the inlet air: 21, 11, 8). Bottom rows: values of the carbon balance mismatch in percentage including or excluding arabinitol. OUR, oxygen uptake rate; CER, CO_2 _exchange rate.

### Identification of the unknown extracellular metabolite

To identify the unknown extracellular compound, a hypoxic culture under steady state conditions was fed with fresh medium containing a carbon source mixture consisting of 12% (w/w) of uniformly ^13^C-labeled glucose and 88% (w/w) of unlabeled glucose, as described in the materials and methods section. Filtered samples of output culture media were submitted to an NMR analysis in order to identify the unknown compound. The NMR results (Additional file 4) were compared with the ^13^C-NMR Japanese database spectra SBDS (Spectral Database for Organic Compounds, http://riodb01.ibase.aist.go.jp/sdbs/cgi-bin/cre_index.cgi?lang=eng). By combining this information with the molecular weight determined by LC-MS and its retention time in HPLC analysis (Additional file 3), the unknown metabolite was identified as arabinitol (MW 152).

Arabinitol, a C5 sugar alcohol related to the pentose phosphate pathway, has been previously shown to be produced by a variety of yeast species during fermentation. For instance, glycerol and arabinitol accumulation has been previously observed in *Pichia anomala *cultures during growth in high-salt environments and on highly concentrated sugar substrates by Bellinger and Larher [28] and Tokuoka *et al*. [29]. Passoth and collaborators [30] suggested that arabinitol has the same physiological role as glycerol in the protection to osmotic stress, since they also found a glycerol and arabinitol accumulation under oxygen limiting conditions in *P. anomala*.

In yeast (*S. cerevisiae*), glycerol is produced under oxygen limitation to reoxidize the redox equivalents produced during amino acid synthesis [31]. Arabinitol has not yet been reported to be involved in the redox metabolism during fermentative growth; however, it is possible that arabinitol formation also plays a role in the redox balance of the cells [30].

**Figure 5:**
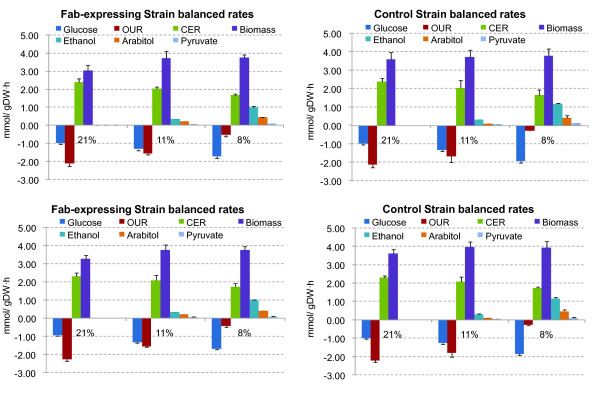
**Substrates consumed and metabolites generated for the different experimental conditions**. Top row: measured values for the Fab-expressing strain (left) and for the control strain (right). Bottom row reconciled values for the Fab-expressing strain (left) and for the control strain (right). Negative values represent substrate consumed, while positive values represent products generated. OUR, oxygen uptake rate; CER, CO_2 _exchange rate.

Once the missing compound was identified, it was quantified by HPLC. Including the arabinitol in the mass balance allowed to significantly improve the carbon balance and the percentage of carbon recovery reached values similar to the ones obtained for the cultivations under normoxic conditions. With this additional analysis, a reconciliation step including all available data was performed resulting in improved consistency index values (h values). Figure 5 shows the measured values, as well as the reconciled values for the involved metabolites in terms of mmol/(gDW· h). As expected, it can be observed that the oxygen consumed per unit of dry weight (DW) decreased when oxygen limitation and hypoxic conditions were applied. Also, the amount of glucose consumed per unit of dry weight is increased. This increased specific consumption of carbon source results mainly in the production of the above mentioned metabolites as a result of the imposed oxygen limitation conditions.

## Conclusions

In this study, different experimental methods were applied in order to obtain first hand data on the biomass composition of *P. pastoris*. The diversity of the data obtained was combined, using *ad hoc *data reconciliation techniques, and the best estimation of the biomass composition was obtained.

Application of the elemental mass balances to the input output metabolite data allowed detecting the lack of one major metabolite. Using complementary NMR data the missing metabolite was identified as being arabinitol. Quantification of this metabolite and its incorporation into the metabolite mass balances allowed to validate the obtained data and to calculate reconciled and consistent fermentation data. The obtained results will be used in metabolic flux analyses of *P. pastoris*, which are being carried out in parallel with transcriptomics and proteomics analyses, in a baseline study aimed at the understanding of the effect of the oxygenation conditions on heterologous protein production in *P. pastoris *.

## Methods

### Strains

In this study, two different strains were used: A *P. pastoris *X-33 (wild type phenotype, Invitrogen) transformed with pGAPα A (Invitrogen) as mock vector (control strain), and a X-33-derived strain expressing the human antibody fragment (Fab) 3H6 fragment under the transcriptional control of glyceraldehyde-3-phosphate dehydrogenase (GAP) constitutive promoter, using the secretion signal sequence from the *S. cerevisiae *α mating factor (Fab-expressing strain) [14].

### Cultivation conditions

Chemostat cultivations were performed in a 2-liter bench-top bioreactor (B. Braun Biotech International, Biostat B), as previously described [16]. Briefly, cells were grown under carbon-limited conditions at a dilution rate (*D*) of 0.1 h^-1^, with different oxygen concentrations in the bioreactor inlet air. The total inlet gas flow was controlled by mass flow meters (Bronkhorst High-Tech) at 1.5 vvm. The outlet gas flow was dried through two silica gel columns to remove the humidity and analyzed to determine its CO_2 _and O_2 _content (BCP-CO_2 _and BCP-O_2 _Sensors, BlueSens). In the cultures that received less than 21% of O_2 _in the inlet gas stream (that is, 11% and 8% of O_2_), air was mixed with the necessary volume of N_2 _so that the total gas flow of 1.5 vvm was maintained constant. The pressure in the culture vessel was maintained at 1.2 bars using a pressure valve (GO Inc). The pH, stirring speed and temperature were maintained at 5 (with 20% NH_3_), 700 rpm and 25°C, respectively.

During the experiments the data acquisition and control of the different variables was done using UAB proprietary software (O. Cos, Universitat Autònoma de Barcelona). For each independent experiment, chemostat culture conditions were maintained constant for at least 5 residence times to allow reaching a metabolic steady state, except for the hypoxic conditions (8% O_2 _in the inlet air), where a wash out after 3.5 residence times had previously been observed [16]. In this case, samples were taken just after 3 residence times and analyzed for biomass composition. At least three independent samples, from two independent cultivations, were analyzed for each tested condition.

### Biomass Analyses

#### Determination of biomass ash content

Culture samples of 20 ml volume were centrifuged at 5,000 rpm, 4°C for 5 min and the pellets were washed twice in 20 ml of 20 mM Tris· HCl, pH 7.6. The resuspended cells were filtered through pre-dried glass fibre filters (Millipore) and dried overnight at 100°C. Combustion of the pre-dried biomass was done by placing the glass fibre filters in ceramic cups (Millipore) in an oven (Hengstler) at 550°C for 12 h.

#### Biomass lyophilisation

Most of the analyses were performed using lyophilized biomass samples. To lyophilize the biomass a sample of cultivation broth was centrifuged at 13,000 rpm for 1 min and the cell pellet was washed twice with 1 ml of 20 mM Tris· HCl, pH 7.6 to get a pellet free of culture medium. The recovered pellet was immediately frozen by immersion in an acetone-dry ice mixture followed by the lyophilization step under vacuum (Virtys Sentry).

#### Biomass elemental analysis

The elemental composition of the biomass was determined taking 1 mg of a lyophilized biomass, adding 1 mg of V_2_O_5 _and introducing the samples in an oven at 1000°C. The volatile compounds were measured with an Elemental Analyzer (NA2100 ThermoFisher).

#### Biomass amino acid composition

Samples of 40 mg of lyophilized biomass were hydrolyzed with 3 ml 6 M HCl at 105°C for 24 h. A N_2 _stream was used to remove the O_2_, minimizing the oxidation during hydrolysis. Following the hydrolyzation step, the volume of the samples was completed up to 50 ml with deionised water (MilliQ). A volume of 500 μl of the diluted samples and 100 μl of 2500 μM nor-leucin (non-proteinogenic amino acid used as internal standard), were mixed before being evaporated to dryness. The resulting pellets were dissolved with 1 ml buffer solution (lithium-citrate loading buffer pH 2.2, Biochrom) and filtered by ultracentrifugation using 10,000 Da cut-off filter (Microcon YM10, Millipore). A 50 μl volume of the filtrate was injected in an amino acid analyzer (Biochrom 30; using the Biochrom EZ Chrom software, for quantification) using a cation exchange chromatography column and post derivatisation with ninhidrine, (as described by Moore, Spackman, and Stein [32]). For the amino acid identification, the eluent (lithium citrate buffer, Biochrom UK) was heated up to 135°C and mixed with ninhydrine. Identification was done according to the known retention time of the amino acid standards in this conditions and quantification performed according to the previously performed calibration curves relating peak area with concentration (detection limit: 1 nmol, reproducibility: 1.5% for 10 nmol).

#### Biomass total protein content

Total protein content was determined by means of the Lowry method as described in [33] from a solution of lyophilized biomass at 0.5 g/l dry weight. Protein concentration was calculated using bobine serum albumin (BSA) as standard.

#### Biomass total carbohydrates content

Total carbohydrates were determined by the phenol method as described in [33]. A 1 ml sample of lyophilized biomass (0.1 mg dry biomass/ml) was mixed with 1 ml phenol 5% and 5 ml 96% sulphuric acid. After 10 min tubes were cooled (15 min 25°C). Absorbance at 488 nm was measured using glucose solutions as standard. Results were corrected for the presence of nucleic acid pentoses using a relative absorbance of 0.455 AU and 0.264 AU for RNA and DNA, respectively [11].

#### Biomass glycogen content

The glycogen content was analyzed as described in the literature [34]. Glycogen was hydrolyzed adding 10 ml of 0.6 M HCl to 20 mg of freeze dried biomass and incubating in a water bath at 100°C for 1 h. After cooling, samples were filtered through 0.22 μm filter (Millipore). The glucose produced was quantified using an automatic glucose analyzer (YSI 1500, Yellow Springs Instruments).

#### Biomass trehalose content

Determination of trehalose content of the biomass was performed according to [35].

10 mg of lyophilized biomass was resuspended in 5 ml water and incubated for 15 minutes in a water bath. After cooling and centrifugation the cellular extract was rinsed twice with water and resuspended in 0.1 M acetate buffer pH 4.5. A sample of 800 μl of the washed cellular extract was mixed with 200 μl of a threhalase (Sigma) solution (1.37 μU/μl in acetate buffer pH 4.5) and incubated overnight. The glucose released was determined using a glucose oxidase kit (Sigma) using the same reaction mixture without threhalase as a blank.

#### Biomass lipid content

Lipid extraction was performed according to [36]. Briefly, 150 mg of freeze dried biomass were extracted with hexane:isopropanol (3:2 v:v) overnight. After adding 0.47 M Na_2_SO_4 _phases were separated with the aid of a centrifugation step. The hexane phase was recovered and evaporated to dryness under a N_2 _flow. The increase in dry weight of the tube was taken as total lipids. Pellet was redissolved in chloroform and further analyzed by thin layer chromatography using silica gel plates (Merck) and a mobile phase of chloroform:methanol:water:acetic acid (345:133:21:3). A standard was analyzed in parallel containing phosphatidic acid (Sigma), phosphatidyl serine (bovine, Fluka), phosphatidyl glycerol (bovine, Sigma), cardiolipine (bovine, sigma), phosphatidyl ethanolamine (bovine, sigma), phosphatidil inositol (bovine, sigma), phosphatidyl coline (bovine, sigma), ergosterol (Fluka) and triacilglycerides (Sigma) using a range of concentrations from 1 to 8 μg of each standard. After running the chromatography the plates were stained either with iodine or with Sudan Black (Sebia). The developed images were quantified using an image analysis software (Multi gauge, Fujifilm) comparing the intensity of the stains with the standards.

#### Biomass DNA content

DNA content of biomass was determined by means of the Hoechst fluorescent dye method as described in [37]. In short, lyophilized biomass samples were dissolved in TNE buffer (1 M NaCl, 10 mM EDTA, 0.1 M Tris· HCl, pH 7.4) at a concentration of 25 mg/ml. Sample solution was mixed with 2 ml of the Hoechst dye solution (Hoechst 33258, 0.5 μg/ml in TNE buffer). Fluorescence was measured using the excitation/emission wavelengths of 356/468. DNA content was calculated by interpolation in a calibration curve performed using standard DNA (DNA sodium salt from calf thymus from Sigma-Aldrich).

#### Biomass RNA content

RNA content of biomass was determined according to Benthin [38]. Briefly 5 mg of lyophilized biomass were resuspended in 10 ml of cold 0.7 M HClO_4 _and incubated for 5 min. After incubation biomass was centrifuged (8,000 rpm, 10 min, 4°C), washed twice and resuspended in 10 ml 0.3 M KOH. Two 5 ml aliquots of the resuspended biomass were incubated at 37°C for 1 h. After cooling 1 ml cold 3 M HClO_4 _was added and samples were centrifuged in the same conditions. Supernatant was collected and the pellet washed twice with 1 ml 0.5 M HClO_4_. The 3 supernatants collected were mixed and absorbance measured at 260 nm in a quartz cuvette. The percentage (w/w) was calculated using 1 A_260_unit (1 cm path length): 0.038 mgRNA/ml and taking into account the sample dilution.

#### Fab quantification by ELISA

Fab amounts in soluble cell extracts and in culture broths were performed by means of a sandwich ELISA assay, as previously described [14]. For the quantification of the intracellular Fab cells were disrupted using a cell disruptor (One Shot. Constant Cell Distruption System LTD). Briefly, 5 ml of 10 g/l of lyophilized biomass resuspended in PBS buffer supplemented with 2% (w/v) SDS, Complete™ protease inhibitor cocktail (Roche) and 0.1% βα-mercaptoethanol, were disrupted at 2 Kbar of pressure in one shot. Fab analysis was performed as described for soluble cell extracts.

#### Bioreactor culture broth concentrations

Cell biomass was monitored by measuring the optical density at 600 nm (OD_600_). For cellular dry weight, a known volume of cultivation broth was filtered using pre-weighted filters; these were washed with two volumes of distilled water and dried to constant weight at 105°C for 24 h. Samples for extracellular metabolite analyses were centrifuged at 10,000 rpm for 2 min in a microcentrifuge to remove the cells and subsequently filtered through 0.45 μm-filters (Millipore type HAWP). Glucose, organic acids, ethanol and arabinitol were analyzed by HPLC (Series 1050, Hewlett Packard) with an ionic exchange column (Bio-Rad, Aminex HPX-87H). As mobile phase, 15 mM sulphuric acid was used. The metabolites were detected (HP 1047A, Detector IR HP, Hewlett Packard) and quantified with the Empower-Profor Software.

#### Identification of extracellular metabolites

##### LC-MS analyses

Extracellular metabolites were identified by means of LC-MS. For this purpose, a volume of about 50 ml of cleared culture supernatant was filtered through a 0.22 μm Millipore filter. The analysis of filtered supernatants were performed on a Shimadzu Prominence HPLC with a UV/VIS detector coupled to a Mass Spectrometry detector Shimadzu 2010A equipped with an ESI (Electro Spray Ionization) interface operating at a wavelength of 210 nm.

Metabolite compounds were separated on a 300 mm × 7.8 mm Aminex HPX-87H column (Biorad) using 0.15 mM formic acid in miliQ water at pH 3 as mobile phase in isocratic mode. The analyses were performed at a flow rate of 0.6 ml/min at room temperature using a 20 μl injection volume.

##### NMR analyses

To identify the unknown extracellular metabolite by NMR, one of the replica cultivation experiments under hypoxic conditions, was further used to perform a ^13^C-labelling experiment with uniformly ^13^C-labeled glucose (Cortecnet), following a previously reported procedure [39]. In brief, isotopic labelling was achieved by feeding the bioreactor (at steady state conditions) with the medium containing about 10% (w/w) of uniformly ^13^C-labeled and 90% unlabeled substrate for one volume change. After that, a volume of 50 ml of fresh sample from the chemostat culture was centrifuged at 4°C, 5000 rpm for 5 min. The supernatant was filtered through a 0.22 μm Millipore filter, stored overnight at -80°C and subsequently lyophilized. The lyophilisate residue was resuspended in a small volume of D_2_O prior to NMR analysis.

^13^C NMR spectra of samples resuspended in D_2_O were analyzed using a Bruker Avance 500 MHz spectrometer, operating at 125 MHz for ^13^C, equipped with a cryoprobe and with about one hour accumulation. Spectra were referenced against an external standard in order to preserve sample recovery.

##### Numerical calculations

Al numerical calculations were performed using Matlab 2007b.

## Competing interests

The authors declare that they have no competing interests.

## Authors' contributions

MC performed bioreactor cultivations, cultivation experimental data acquisition and biochemical analyses, data calculation, analysis and interpretation of results and participated in drafting the manuscript. IT participated together with MC and KB in performing bioreactor cultivations, obtaining and analyzing bioreactor experimental data. KB participated in the experimental design of bioreactor cultivations, cultivation, cultivation analyses, arabinitol identification and interpretation of results. FS performed spectrometry analysis. DM participated in conceptual and experimental design of bioreactor cultivations. PF participated in the overall conceptual and experimental design of this study, interpretation of results and in drafting the manuscript. JA participated in the overall conceptual and experimental design of this study, data calculation, analysis and interpretation of results and in drafting the manuscript. All authors revised and approved the final manuscript.

## Supplementary Material

Additional file 1 **Measured chemical elements in the biomass**. Major chemical elements measured in the biomass for each strain at the different experimental conditions (% O_2 _in the inlet air: 21, 11, 8). Data given as percentage of dry weight. Click here for file

Additional file 2 **Calculated C-mol formula for each biomass component**. Calculated C-mol formulas of the molecular biomass components at the different experimental conditions (% O_2 _in the inlet air: 21, 11, 8). * Data from Lange and Heijnen 2001 [11] for *S. cerevisiae*. Click here for file

Additional file 3 **HPLC chromatogram of a hypoxic culture broth**. Chromatogram with the unknown peak at retention time of 11.5 min in a HPLC. Hewlett Packard 1050 Biorad Aminex HPX-87C ion-exchange resin column. Click here for file

Additional file 4 **NMR spectrum of a hypoxicculture broth**. 13 C NMR spectrum of a D_2_O-resuspended lyophilised hypoxic culture broth sample in a Bruker Avance 500 MHz spectrometer. Click here for file
