# Supplementary material for: Macromolecular and elemental composition analysis and extracellular metabolite balances of Pichia pastoris growing at different oxygen levels
Source: Microb Cell Fact. 2009 Dec 9;8:65. doi: 10.1186/1475-2859-8-65 (PMC2799386; doi:10.1186/1475-2859-8-65)
Supplement: Additional file 1 — Measured chemical elements in the biomass. Major chemical elements measured in the biomass for each strain at the different experimental conditions (% O2 in the inlet air: 21, 11, 8). Data given as percentage of dry weight. [file 1475-2859-8-65-S1.PDF]

Macromolecular and elemental composition analysis and extracellular metabolite balances of *Pichia pastoris* growing at different oxygen levels

Marc Carnicer[1], Kristin Baumann[1], Isabelle Töplitz[1,4*], Francesc Sánchez-Ferrando[2], Diethard Mattanovich[3,4], Pau Ferrer[1], Joan Albiol[1§]

Additional file 1 – Measured chemical elements in the biomass.

	Fab-expressing strain						Control strain					
	21 %		11 %		8 %		21 %		11 %		8 %	
	% w/w	sd	% w/w	sd	% w/w	sd	% w/w	sd	% w/w	sd	% w/w	sd
C	43.7	2.2	44.4	2.2	44.3	2.2	42,7	2.1	43.6	2.2	44.4	2,2
H	6.8	0.3	6.8	0.3	6.8	0.3	6.7	0.3	6.8	0.3	7.0	0,3
N	7.1	1.1	6.9	1.0	7.5	1.1	7.2	1.1	7.1	1.1	7.0	1,0
S	0.2	0.1	0.2	0.1	0.1	0.1	0.2	0.1	0.2	0.1	0.2	0,1
Ash	7.2	0.4	7.2	0.4	7.2	0.4	7.2	0.4	7.2	0.4	7.2	0,4

Measured chemical elements in the biomass.

Major chemical elements measured in the biomass for each strain at the different experimental conditions (% O_2 in the inlet air: 21, 11, 8). Data given as percentage of dry weight.
