# Supplementary material for: Macromolecular and elemental composition analysis and extracellular metabolite balances of Pichia pastoris growing at different oxygen levels
Source: Microb Cell Fact. 2009 Dec 9;8:65. doi: 10.1186/1475-2859-8-65 (PMC2799386; doi:10.1186/1475-2859-8-65)
Supplement: Additional file 2 — Calculated C-mol formula for each biomass component. Calculated C-mol formulas of the molecular biomass components at the different experimental conditions (% O2 in the inlet air: 21, 11, 8). * Data from Lange and Heijnen 2001 [11] for S. cerevisiae. [file 1475-2859-8-65-S2.PDF]

Macromolecular and elemental composition analysis and extracellular metabolite balances of *Pichia pastoris* growing at different oxygen levels

Marc Carnicer[1], Kristin Baumann[1], Isabelle Töplitz[1,4*], Francesc Sánchez-Ferrando[2], Diethard Mattanovich[3,4], Pau Ferrer[1], Joan Albiol[1§]

Additional file 2 – Calculated C-mol formula for each biomass component.

Saccharomyces cerevisiae (*)	
Protein	$C\ H_{1.581}\ N_{0.275}\ O_{0.318}\ S_{0.003}$
Carbohydrate	$C\ H_{1.667}\ O_{0.833}$
Lipids	$C\ H_{1.873}\ N_{0.010}\ O_{0.149}\ P_{0.010}$
RNA	$C\ H_{1.232}\ N_{0.389}\ O_{0.737}\ P_{0.105}$
DNA	$C\ H_{1.255}\ N_{0.378}\ O_{0.312}\ P_{0.102}$

21 % Oxygen setpoint	
Protein	$C\ H_{1.503}\ N_{0.259}\ O_{0.347}\ S_{0.002}$
Carbohydrate	$C\ H_{1.667}\ O_{0.833}$
Lipids	$C\ H_{1.697}\ N_{0.000}\ O_{0.156}\ P_{0.004}$
RNA	$C\ H_{1.230}\ N_{0.400}\ O_{0.734}\ P_{0.105}$
DNA	$C\ H_{1.232}\ N_{0.390}\ O_{0.632}\ P_{0.105}$

11 % Oxygen setpoint	
Protein	$C\ H_{1.512}\ N_{0.256}\ O_{0.344}\ S_{0.002}$
Carbohydrate	$C\ H_{1.667}\ O_{0.833}$
Lipids	$C\ H_{1.746}\ N_{0.000}\ O_{0.145}\ P_{0.003}$
RNA	$C\ H_{1.230}\ N_{0.400}\ O_{0.734}\ P_{0.105}$
DNA	$C\ H_{1.232}\ N_{0.390}\ O_{0.632}\ P_{0.105}$

8 % Oxygen setpoint	
Protein	$C\ H_{1.511}\ N_{0.255}\ O_{0.340}\ S_{0.002}$
Carbohydrate	$C\ H_{1.667}\ O_{0.833}$
Lipids	$C\ H_{1.780}\ N_{0.000}\ O_{0.144}\ P_{0.002}$
RNA	$C\ H_{1.230}\ N_{0.400}\ O_{0.734}\ P_{0.105}$
DNA	$C\ H_{1.232}\ N_{0.390}\ O_{0.632}\ P_{0.105}$

Biomass constituent C-mol formulas depending on the oxygenation condition

Calculated C-mol formulas of the molecular biomass components at the different experimental conditions (% O_2 in the inlet air: 21, 11, 8). * Data from Lange and Heijnen 2001 [11] for *S. cerevisiae*.
