# Supplementary material for: Macromolecular and elemental composition analysis and extracellular metabolite balances of Pichia pastoris growing at different oxygen levels
Source: Microb Cell Fact. 2009 Dec 9;8:65. doi: 10.1186/1475-2859-8-65 (PMC2799386; doi:10.1186/1475-2859-8-65)
Supplement: Additional file 3 — HPLC chromatogram of a hypoxic culture broth. Chromatogram with the unknown peak at retention time of 11.5 min in a HPLC. Hewlett Packard 1050 Biorad Aminex HPX-87C ion-exchange resin column. [file 1475-2859-8-65-S3.PDF]

Macromolecular and elemental composition analysis and extracellular metabolite balances of *Pichia pastoris* growing at different oxygen levels

Marc Carnicer[1], Kristin Baumann[1], Isabelle Töplitz[1,4*], Francesc Sánchez-Ferrando[2], Diethard Mattanovich[3,4], Pau Ferrer[1], Joan Albiol[1§]

Additional file 3 – HPLC chromatogram of a hypoxic culture broth.

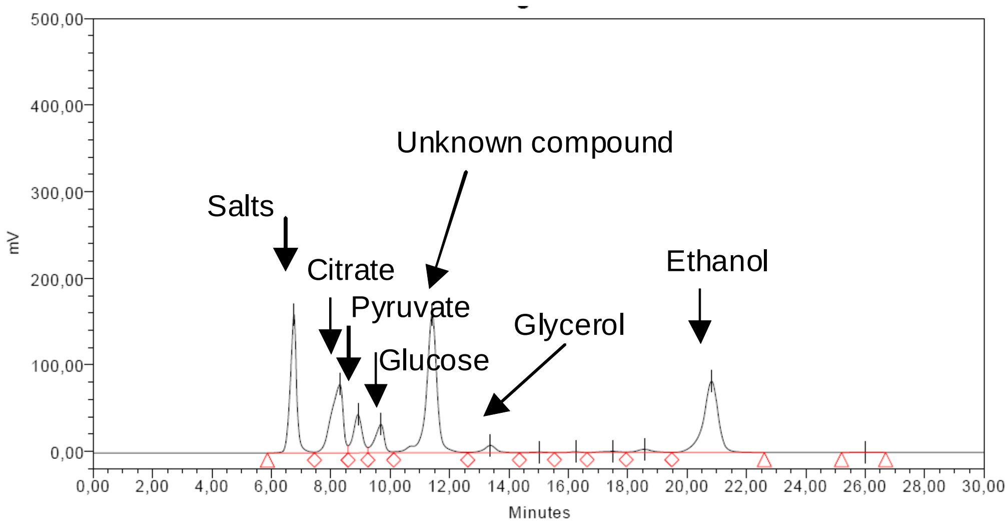

Chromatogram with the unknown peak at retention time of 11.5 min in a HPLC Hewlett Packard 1050 Biorad Aminex HPX-87C ion-exchange resin column
