# Supplementary material for: Macromolecular and elemental composition analysis and extracellular metabolite balances of Pichia pastoris growing at different oxygen levels
Source: Microb Cell Fact. 2009 Dec 9;8:65. doi: 10.1186/1475-2859-8-65 (PMC2799386; doi:10.1186/1475-2859-8-65)
Supplement: Additional file 4 — NMR spectrum of a hypoxicculture broth. 13 C NMR spectrum of a D2O-resuspended lyophilised hypoxic culture broth sample in a Bruker Avance 500 MHz spectrometer. [file 1475-2859-8-65-S4.PDF]

Macromolecular and elemental composition analysis and extracellular metabolite balances of *Pichia pastoris* growing at different oxygen levels

Marc Carnicer[1], Kristin Baumann[1], Isabelle Töplitz[1,4*], Francesc Sánchez-Ferrando[2], Diethard Mattanovich[3,4], Pau Ferrer[1], Joan Albiol[1§]

Additional file 4 – NMR spectrum of a hypoxic culture broth

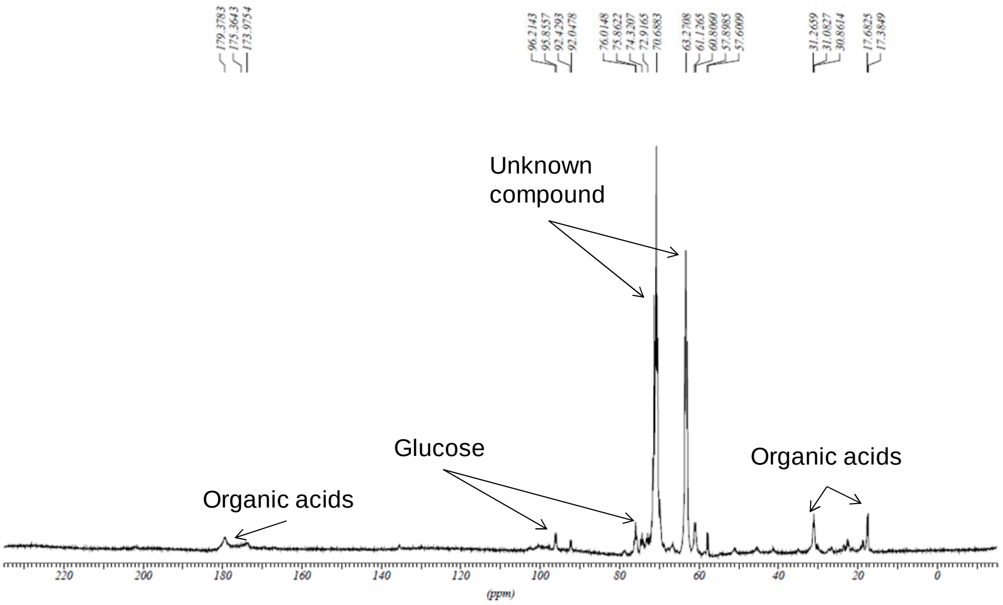

^{13}C NMR spectrum of a D_2O-resuspended lyophilised hypoxic culture broth sample in a Bruker Avance 500 MHz spectrometer.
